# Association between dental caries and adherence to the Mediterranean diet, dietary intake, and body mass index in children

**DOI:** 10.1186/s12903-024-04020-3

**Published:** 2024-03-02

**Authors:** Kübra Esin, Beyza Ballı-Akgöl, Saniye Sözlü, Betul Kocaadam-Bozkurt

**Affiliations:** 1 Faculty of Health Sciences, Department of Nutrition and Dietetics, Tokat Gaziosmanpaşa University, Tokat, Türkiye; 2 School of Dentistry, Department of Pediatric Dentistry, Antalya Bilim University, Antalya, Turkey; 3 Faculty of Health Sciences, Department of Nutrition and Dietetics, Erzurum Technical University, Erzurum, Türkiye

**Keywords:** Dental caries, Children, Diet quality, Mediterranean Diet

## Abstract

**Background:**

Children with healthier nutritional status are less likely to develop severe caries than those with a high-sugar content diet. Studies evaluating dental caries and nutritional status in school-age children have generally focused on dietary intake, diet quality, or anthropometric measures, and the number of studies evaluating them together is limited.

**Objective:**

It was aimed to evaluate the relationship between dental caries adherence to the Mediterranean Diet (MD), dietary intake, and Body Mass Index (BMI) in school-age children.

**Materials and methods:**

This study was conducted with 300 healthy children (52.0% boys, 48.0% girls) aged between 6 and 12 years. The data collection forms included sociodemographic characteristics, oral health practices of children, Mediterranean Diet Quality Index for children and adolescents (KIDMED), and food consumption records. Anthropometric measurements (body weight and height) of the children were taken. Dental examinations were performed by a pediatric dentist.

**Results:**

While the DMFT mean score of the children was 1.7 ± 2.09, the mean dft score was 2.9 ± 3.29. The mean of KIDMED scores was 5.9 ± 3.32. DMFT and dft scores decreased statistically as maternal education increased (*p* < 0.05). DMFT and dft scores were not statistically different between BMI groups according to gender and age (*p* > 0.05). DMFT scores differed statistically between KIDMED groups (*p* < 0.05). This difference was between low-optimal and low-improvement-needed groups. While there was a low negative correlation (*r*=-0.169) between calcium intake and DMFT score, a low positive correlation was found between glucose (*r* = 0.172) and fructose (*r* = 0.149) intake and dft score (*p* < 0.05). In regression analysis, while the children’s age related DMFT scores positively, maternal education and KIDMED scores related DMFT scores negatively. Also, children’s age and maternal education related dft scores negatively.

**Conclusion:**

In this study, adherence to the MD rather than nutrients was found to be important in dental caries. Also maternal education level was also found to be a determinant factor in dental caries in children. DMFT and dft did not differ between BMI groups.Further studies should be conducted to assess the impact of the MD on dental caries in children to develop dietary interventions for preventative purposes.

## Introduction

Dental decay (also known as caries or cavities) is one of the most common chronic diseases in children [1, 2]. Worldwide, 53.8% of children have permanent tooth decay, and 46.2% have primary tooth decay [3]. According to the Turkey Oral and Dental Health Profile 2018 study, it was determined that 64.4% of children aged 5 years, 46.6% of children aged 12 years, and 58.3% of children aged 15 years had at least one tooth decayed [4]. Dental caries in children negatively affects their quality of life due to discomfort, pain, sleep problems, chewing difficulties, learning disorders, and absenteeism from school [5].

Dental caries is a multifactorial disease. The interaction of environmental and genetic factors, such as the effects of oral bacterial flora, dietary habits, oral hygiene, saliva composition, and tooth structure, plays a role in the formation and progression of caries [6]. The eating pattern has an important role in the etiology of dental caries, as there is a multifaceted relationship between diet and dental caries among these factors. Eating habits can be a risk factor or preventive for dental caries. On the other hand, impaired oral health can lead to insufficient dietary intake and nutrient deficiencies [7].

Over the past decade, there has been a growing interest in investigating the potential preventive effects of a healthy diet against developing dental caries in children [8]. Children with healthier eating habits are less likely to have dental caries than those with unhealthy ones [9, 10]. The type, physical and chemical structure, preparation, consumption amount, and frequency of the foods consumed in children affect the formation of dental caries [11]. Dietary free sugars are the leading risk factor for developing dental caries [12]. Studies have shown that 100% fruit juice consumption, sugar consumption more than once a week, consumption of processed starch, and consumption of sugary drinks at bedtime are associated with a higher risk of dental caries in children [13]. American Academy of Pediatric Dentistry recommend reducing sugar consumption to less than 10% of total energy intake and reducing sugar intake to less than 5% to reduce body weight gain, obesity and the risk of dental caries in children [14].

Among healthy dietary patterns, the Mediterranean diet (MD) draws attention. The MD is characterized by a high intake of plant-based foods such as vegetables, fruits, nuts, legumes, and unprocessed cereals. It also involves a moderate to high consumption of fish, while limiting the intake of meat, particularly red and processed meats. Polyols, also known as polyalcohols, are naturally occurring compounds present in plant-based foods including fruits and vegetables. These substances are recognized for their anticariogenic properties, since they promote a rise in salivary pH. Consequently, they facilitate the remineralization of early-stage carious lesions [15, 16]. Moreover, the presence of phenolic compounds in fruits and vegetables has been seen to inhibit the proliferation of bacteria associated with dental caries and the development of biofilm. Additionally, these compounds have been reported to have inhibitory properties against glycosyltransferases, which are enzymes responsible for facilitating the metabolic breakdown of sucrose [17, 18].

In addition, the MD largely excludes ultra-processed food, carbonated beverages, and manufactured pastries due to their high content of added sugars. The high sugar content foods, beverages, and starchy foods, particularly those more rapidly digested in the oral cavity, drop plaque pH and may pose an increased risk of dental caries. Many foods that are at the core of theMD have a low glisemic index (GI), and this could play a role in the salutary effects of this dietary model [19]. Lower GI foods, which a more slowly digested, showing smaller dental pH excursions, and potentially lead to an decreased risk of dental caries formation in the longer term [20]. Evidence supports the association between dietary patterns and dental caries; however, research on the association between dental caries and the MD is limited [21, 22].

The relationship between caries and obesity has received much attention in recent years [23]. The association is likely derived from common risk factors such as high sugar diet, unhealthy eating habits, lower socio-economic status, and other social-environmental factors [24]. Hayden et al. reported that a significant association between children obesity and dental caries only in industrialised countries [25]. Hooley et al. reported that dental caries is associated with both high and low body mass index [26]. Recent systematic reviews [27, 28] showed that there is no evidence of a consistent association between BMI and caries.

Considering that school age is a compelling period in which every child’s health-related behaviors, beliefs, and attitudes become widespread, determining the nutritional status of children that predispose/resist tooth decay in this period constitutes a critical period to prevent dental caries [29]. Studies evaluating dental caries and nutritional status in school-age children have generally focused on dietary intake, diet quality, or anthropometric measures, and the number of studies evaluating them together is limited. This study assessed the relationship between dental caries and adherence to the MD, dietary intake, and BMI in school-age children.

## Materials and methods

### Study setting and population

This study was conducted with 300 healthy children (52.0% boys, 48.0% girls) aged between 6 and 12 years (10.2 ± 1.84 years) who applied to a private dental clinic in the province of Istanbul. The G*Power (version 3.1.9.7, Universitat Düsseldorf, Düsseldorf, Germany) program was used for post-hoc power analysis after the study, with correlation: point biserial model, according to the with two tail, effect size |ρ|=0.33, α err prob = 0.05, and the power (1-ß err prob) of the study was determined to 99%. Children who do not have a chronic/ psychological problem affecting their nutrition or require special nutrition, and who agreed to participate in the study were included in the sampling (response rate and any data about non-participants are not available).

Ethical approval was obtained from the Istanbul Medipol University Ethics Committee (10840098-604.01.01-E.27,469) to conduct the research. The research complied with the policy set out in the Declaration of Helsinki; informed consent in written form was obtained from the parents, and child consent was obtained.

### Measures

Data on children and their parents were collected through face-to-face interviews with parents using a questionnaire form. The data collection forms included sociodemographic characteristics [children’s age, sex (male or female), educational status of children (elemantary or secondary school) and educational status of parents (< high school or ≥ high school) etc.], health information (a disease diagnosed by a doctor, yes or no), oral health practices of children (toothbrushing status, daily frequency of tooth brushing), Mediterranean Diet Quality Index for children and adolescents (KIDMED), and food consumption records. Anthropometric measurements of the children were taken by the researcher (K.E). The oral examinations of the children were made by the pediatric dentist (B.B.A).

### Dental examination

In order to evaluate the dental health practices of the children, the use of toothbrushes, the status and frequency of tooth brushing were questioned with a questionnaire. Clinical examinations were made by the pediatric dentist.

Oral and dental examinations of children were performed with the help of mirror and probe according to the World Health Organization(WHO) criteria [30]. Radiography was not taken to determine interproximal caries. If any surface of the tooth became visible in the mouth, the tooth was considered erupted. If both primary and permanent teeth were in the same tooth space at the same time, permanent teeth were included in the evaluation. Teeth with no clinically carious cavities on the surface of the tooth were considered healthy. If there were no other pathological findings, white spots, hard discolorations and pits on the enamel surface were included in the same classification as healthy. WHO recommended caries classification was used and only cavitated dentine lesions were included [30]. Decayed, missing and filled teeth were calculated according to DMFT ( Decayed, Missing and Filled Teeth) index for permanent dentition, dft ( decayed and filled teeth) index for primary dentition [30]. Because the included children were at the age of mixed dentition, the missing “m” component of the dmft was not included.

### Dietary intake

Children’s food consumption records were obtained with the help of their parents by the researchers. The children and their parents were instructed on how to record food consumption and were asked to record all foods and drinks consumed for two days (one day weekday and one weekend day) by the researchers. A “Meal and Food Photo Catalog” was used to determine the sizes and amounts of the food and beverages consumed [31]. The energy and nutrient intakes (protein, fat, carbonhydrate, glucose, fructose, galactose, sucrose, fiber, calcium, floridine, iodine) [32, 33] of children were evaluated using the Nutrition Information System (BeBiS) program (version 8) [34]. The children’s adherence to the MD was assessed using the Mediterranean Diet Quality Index for children and adolescents (KIDMED). The KIDMED was developed by Serra-Majem et al. (2004) to assess adherence to MD in children and adolescents [35]. The Turkish reliability and validity study of the scale was conducted by Şahingöz et al. (2019) [36]. The index ranged from 0 to 12 and was based on 16 questions. The 6th, 12th, 14th, and 16th items are scored as -1, and the remaining 12 items are scored as + 1. The total score is calculated by summing these values:≥8, optimal Mediterranean diet; 4–7, improvement needed; ≤3, low diet quality [35].

### Anthropometric measurements

The researcher performed the measurements of weight using a Tanita BC532 device. A stadiometer was used to measure height [37]. The World Health Organization WHO (2007) growth standards and the WHO AnthroPlus software (version 1.0.4, February 2011) program were used to evaluate Body Mass Index (BMI) Z-scores. The BMI was categorized according to the Z-score junctions [38].

### Statistical analysis

The data was analyzed using the SPSS 22.0. The variables were evaluated using visual (histogram and probability graphs) and skewness and kurtosis to determine whether or not they are normally distributed. In the statistical evaluation of the data continuous variables were expressed as mean ($\bar {x}$) and standard deviation (SD), and categorical variables were expressed as number (n) and percentage (%). Mann Whitney U or Kruskall Wallis tests were applied to find value differences between groups. The Pearson or Spearman correlation coefficient was used to analyze the parameters’ correlation. Multiple linear regression analyses were used to evaluate factors associated with DMFT and dft scores. In the regression analyses, we selected the variables that showed a significant difference according to group comparison or correlation with DMFT or dft as independent variables. The statistical significance level was set at *p* < 0.05.

## Results

Of the children participating in the study, 52.0% were boys and 48.0% were girls. While 50.0% of children had normal BMI, 38.0% were overweight or obese (Table 1). While the DMFT mean score of the children was 1.7 ± 2.09, the mean dft score was 2.9 ± 3.29. The mean of KIDMED scores was 5.9 ± 3.32. Daily dietary energy (kcal), calcium (mg), glucose (g), and fructose (g) intake were 1607.4 ± 445.87, 691.0 ± 319.19, 9.8 ± 7.88 and 11.2 ± 9.35, respectively (Table 2). According to the KIDMED classification, 48.0% of the children needed improvement, 21.3% were in the low diet quality group, and 30.7% were in the optimal MD group. Those who brush their teeth all the time constituted 56.0% of the study, while 65.7% declared that they brushed their teeth once daily. While 65.7% of mothers were high school /university graduates, this rate was 80% for fathers (Table 1).

**Table 1:** General characteristics and KIDMED classification of the participants (n:300)

	n	%
**Children’s age**		
6–9 years	107	35.7
10–12 years	193	64.3
**Sex**		
Boy	156	52.0
Girl	144	48.0
**Children’s education**		
Elementary school	107	35.7
Secondary school	193	64.3
**Children’s BMI classification (total)**		
Underweight	36	12.0
Normal	150	50.0
Overweight	65	21.7
Obese	49	16.3
**Children’s BMI classification (boy)**		
Underweight	15	9.6
Normal	87	55.8
Overweight	30	19.2
Obese	24	15.4
**Children’s BMI classification (girl)**		
Underweight	21	14.6
Normal	63	43.8
Overweight	35	24.3
Obese	25	17.4
**Tooth brushing**		
Sometimes	132	44.0
Always	168	56.0
**Frequency of tooth brushing**		
Once a day	197	65.7
Twice or three times a day	103	34.3
**Mother’s age (years)**		
25–34	85	28.3
35–44	185	61.7
45+	30	10.0
**Father’s age (years**)		
25–34	30	10.0
35–44	196	65.3
45+	74	24.7
**Mother’s education**		
<High school	103	34.3
≥ High school	197	65.7
**Father’s education**		
<High school	60	20.0
≥ High school	240	80.0
**KIDMED**		
Optimal	92	30.7
Improvement needed	144	48.0
Low	64	21.3
	$\bar {X}$ ±**SD**	
**Children’s age (years)**	10.2 ± 1.84	
**Mother’s age (years)**	37.4 ± 5.44	
**Fathers’s age (years)**	41.0 ± 6.10	

**Table 2:** Dental health indices, KIDMED scores, dietary energy and nutrient intake of the participants (n:300)

	Mean ± SD	1st quartile	Median	3rd quartile
**Dental health indices**				
DMFT	1.7 ± 2.09	< 0.001	1.0	3.0
dft	2.9 ± 3.29	< 0.001	2.0	5.0
**KIDMED scores**	5.9 ± 3.32	4.0	6.0	8.0
**Dietary energy (kcal)**	1607.4 ± 445.87	1278.6	1585.6	1886.5
**Protein (g)**	61.2 ± 20.02	48.2	58.5	73.0
**Fat (g)**	69.9 ± 23.86	53.4	65.9	84.5
**Carbohydrate (g)**	177.9 ± 62.7	134.5	174.1	215.6
**Glucose (g)**	9.8 ± 7.88	3.9	8.3	12.7
**Fructose (g)**	11.2 ± 9.35	3.9	9.3	15.4
**Galactose (g)**	1.25 ± 1.25	0.1	1.0	2.0
**Sucrose (g)**	26.1 ± 20.75	9.9	22.1	38.1
**Fiber (g)**	15.9 ± 6.66	11.8	15.3	19.6
**Calcium (mg)**	691.0 ± 319.19	450.0	683.0	902.6
**Fluorine (mcg)**	393.3 ± 148.62	300.6	375.6	469.1
**Iodine (mcg)**	146.3 ± 67.7	95.2	137.0	188.5

Table 3 shows the comparison of dental health indices by groups. DMFT and dft scores differed according to age groups (6–9 and 10–12 years) (*p* < 0.05). It was determined that DMFT and dft scores decreased statistically as maternal education level increased (*p* < 0.05). According to the frequency of tooth brushing, the DMFT score of those who brush their teeth at most once a day (1.5 ± 1.96) was statistically lower than those who brushed their teeth twice or three times a day (2.1 ± 2.29) (*p* < 0.05), but no difference was found between dft scores. DMFT and dft scores were not statistically different between BMI groups according to sex and age (*p* > 0.05). DMFT scores differed statistically between KIDMED groups. This difference was between low-optimal and low-improvement-needed groups (Table 3).

**Table 3:** Comparison of dental health indices by groups (n:300)

	DMFT			dft		
	Mean ± SD	Median (IQR)	*p*-value	Mean ± SD	Median (IQR)	*p*-value
**Children’s age**						
6–9 years	1.4 ± 2.23	1.0 (< 0.001-2.0)	**0.009**	5.6 ± 3.32	6.0 (3.0–8.0)	**< 0.001**
10–12 years	1.9 ± 1.98	1.0 (< 0.001-4.0)		1.5 ± 2.14	< 0.001 (< 0.001-3.0)	
Sex*						
Girls	1.8 ± 1.86	1.0 (< 0.001-4.0)	0.152	2.8 ± 3.14	2.0 (< 0.001-5.0)	0.668
Boys	1.7 ± 2.29	1.0 (< 0.001-3.0)		3.1 ± 3.43	2.0 (< 0.001-6.0)	
Children’s education*						
Elementary school	1.4 ± 2.23	1.0 (< 0.001-2.0)	**0.009**	5.6 ± 3.32	6.0 (3.0–8.0)	**< 0.001**
Secondary school	1.9 ± 1.98	1.0 (< 0.001-4.0)		1.5 ± 2.14	< 0.001 (< 0.001-3.0)	
Children’s BMI classification (total)**						
Underweight	1.6 ± 1.91	1.0 (< 0.001-3.0)		4.1 ± 3.47	4.0 (< 0.001-6.0)	
Normal	1.9 ± 2.33	1.0 (< 0.001-3.3)	0.535	3.1 ± 3.58	2.0 (< 0.001-6.3)	0.104
Overweight	1.5 ± 1.63	1.0 (< 0.001-3.0)		2.1 ± 2.74	1.0 (< 0.001-4.0)	
Obese	1.6 ± 1.98	1.0 (< 0.001-3.0)		2.7 ± 2.64	2.0 (< 0.001-5.0)	
Children’s BMI classification (boy)**						
Underweight	1.5 ± 2.19	1.0 (< 0.001-2.0)	0.788	3.3 ± 3.43	3.0 (< 0.001-5.0)	0.390
Normal	1.9 ± 2.67	1.0 (< 0.001-3.0)		3.1 ± 3.67	2.0 (< 0.001-6.0)	
Overweight	1.3 ± 1.46	1.0 (< 0.001-3.0)		2.5 ± 3.26	0.5 (< 0.001-4.3)	
Obese	1.2 ± 1.47	0.5 (< 0.001-3.0)		3.7 ± 2.72	4.0 (1.0–6.0)	
Children’s BMI classification (girl)**						
Underweight	1.7 ± 1.73	1.0 (< 0.001-3.5)	0.997	4.7 ± 3.45	4.0 (2.5–7.5)	0.052
Normal	1.8 ± 1.77	2.0 (< 0.001-4.0)		3.1 ± 3.47	2.0 (< 0.001-7.0)	
Overweight	1.6 ± 1.76	1.0 (< 0.001-4.0)		2.8 ± 2.21	2.0 (< 0.001-3.0)	
Obese	1.9 ± 2.34	1.0 (< 0.001-3.0)		2.8 ± 2.20	2.0 (< 0.001-3.5)	
Tooth brushing*						
Sometimes	1.7 ± 1.94	1.0 (< 0.001-3.0)	0.808	3.4 ± 3.46	3.0 (< 0.001-6.0)	0.112
Always	1.7 ± 2.20	1.0 (< 0.001-3.0)		2.6 ± 3.12	1.5 (< 0.001-4.8)	
Frequency of tooth-brushing*						
Once a day	1.5 ± 1.96	1.0 (< 0.001-3.0)	**0.014**	3.1 ± 3.36	< 0.001 (2.0-5.5)	0.161
Twice or three times a day	2.1 ± 2.29	2.0 (< 0.001-4.0)		2.6 ± 3.15	1.0 (< 0.001-5.0)	
Mother’s age (years)**						
25–34	1.9 ± 2.38	2.0 (< 0.001-4.0)	0.310	4.0 ± 3.60	4.0 (< 0.001-7.0)^a^	**0.002**
35–44	1.6 ± 2.00	1.0 (< 0.001-3.0)		2.5 ± 3.10	1.0 (< 0.001-4.0)^b^	
45+	1.8 ± 1.62	1.5 (< 0.001-3.3)		2.8 ± 2.87	2.5 (< 0.001-4.3)^a.b^	
**Father’s age (years**)**						
25–34	2.1 ± 3.08	1.5 (< 0.001-3.0)	0.679	4.7 ± 4.27	4.5 (< 0.001-8.0)	0.064
35–44	1.7 ± 1.96	1.0 (< 0.001-3.0)		2.8 ± 3.19	2.0 (< 0.001-5.0)	
45+	1.6 ± 1.93	1.0 (< 0.001-3.0)		2.7 ± 2.91	2.0 (< 0.001-5.0)	
Mother’s education*						
<High school	2.3 ± 2.39	2.0 (< 0.001-4.0)	**0.000**	3.8 ± 3.89	3.0 (< 0.001-7.0)	**0.008**
≥ High school	1.4 ± 1.85	1.0 (< 0.001-3.0)		2.5 ± 2.83	1.0 (< 0.001-5.0)	
Father’s education*						
<High school	2.3 ± 2.76	2.0 (< 0.001-4.0)	0.063	4.5 ± 3.74	4.0 (1.0–8.0)	**< 0.001**
≥ High school	1.6 ± 1.86	1.0 (< 0.001-3.0)		2.6 ± 3.05	1.0 (< 0.001-5.0)	
KIDMED**						
Optimal	1.2 ± 1.68	< 0.001 (< 0.001-2.0) ^a^	**0.003**	3.2 ± 3.11	3.0 (< 0.001-5.0)	0.207
Improvement needed	1.7 ± 1.91	1.0 (< 0.001-3.0) ^a^		3.0 ± 3.42	2.0 (< 0.001-6.0)	
Low	2.4 ± 2.74	2.0 (0.3-4.0) ^b^		2.5 ± 3.26	< 0.001 (< 0.001-5.0)	

*Mann-Whitney U test; **Kruskall-Wallis test; IQR: inter quartile range. ^a.b^The groups with the same letters within a column are not signifcantly different according to pairwise comparisons. Bold values: *p* < 0.05

The correlation between the daily energy and some nutrient values of children and their DMFT and dft scores are shown in Table 4. While there was a low negative correlation (*r*=-0.169) between calcium intake and DMFT score, a low positive correlation was found between glucose (*r* = 0.172) and fructose (*r* = 0.149) intake and dft score (*p* < 0.05). No correlation was found between the intake of energy and other nutrients and DMFT and dft scores (*p* > 0.05).

**Table 4:** Correlation between energy and nutrient intake and Dental health indices

	DMFT		dft	
	r	*p*	r	*p*
Dietary energy (kcal)	0.076	0.291	-0.124	0.083
Protein (g)	-0.007	0.917	-0.140	0.050
Fat (g)	-0.013	0.852	-0.069	0.336
Carbohydrate (g)	0.096	0.182	-0.071	0.321
Glucose (g)	-0.093	0.192	0.172	0.016*
Fructose (g)	-0.083	0.249	0.149	0.036*
Galactose (g)	-0.020	0.781	-0.093	0.194
Sucrose (g)	0.021	0.770	0.108	0.131
Fiber (g)	0.011	0.875	-0.032	0.655
Calcium (mg)	-0.169	0.017*	0.060	0.403
Fluorine (mcg)	-0.114	0.112	-0.011	0.881
Iodine (mcg)	-0.097	0.174	-0.040	0.576

When the factors related the scores obtained from DMFT were evaluated with multiple linear regression analysis (R^2^: 0.113, *p* < 0.001), while the children’s age related DMFT scores positively, maternal education and KIDMED scores related DMFT scores negatively (Table 5). Also, children’s age and maternal education related dft scores negatively (R^2^: 0.454, *p* < 0.001) (Table 5).

**Table 5:** Multiple linear regression analysis for children’s DMFT and dft scores

Model	Unstandardized B	Standart Error	Standardized Coefficients Beta	t	*p*	Lower Bound	Upper Bound
**DMFT**							
Age (years)	0.170	0.064	0.151	2.633	0.009*	0.043	0.297
Mather’s education	-0.827	0.252	-0.188	-3.280	0.001*	-1.323	-0.331
Frequency of tooth-brushing (daily)	0.173	0.238	0.041	0.728	0.467	-0.295	0.641
KIDMED scores	-0.085	0.037	-0.135	-2.305	0.022*	-0.157	-0.012
Dietary Ca intake (mg)	< 0.001	< 0.001	-0.021	-0.361	0.718	-0.001	0.001
		**R**^**2**^:**0.113** ***p*** < **0.001**					
**dft**							
Age (years)	-1.154	0.085	-0.648	-13.599	< 0.001*	-1.321	-0.987
Maternal age	0.028	0.028	0.046	0.988	0.324	-0.028	0.083
Mathers education	-0.864	0.335	-0.125	-2.580	0.010*	-1.523	-0.205
Father’s education	-0.380	0.409	-0.046	-0.928	0.354	-1.186	0.426
Dietary glucose intake (g)	-0.014	0.063	-0.034	-0.225	0.822	-0.139	0.110
Dietary fructose intake (g)	0.017	0.053	0.049	0.324	0.746	-0.087	0.122
		**R**^**2**^:**0.454** ***p*** < **0.001**					

## Discussion

The results of this study show that child age, maternal education level, and KIDMED score were related factors for the DMFT score. Also, it was found that the child’s age and the mother’s educational status were related factors for the dft scores. Although there were relationships between dietary calcium intake, tooth brushing frequency and DMFT score, dietary glucose and fructose intake, and dft score, these relationships were insignificant in the regression models. It was determined that BMI did not associate with the dental health indices.

The World Health Organization (WHO) classified dental caries into 5 groups as very low (0.0-1.1 DMFT), low (1.2–2.6 DMFT), medium (2.7–4.4 DMFT), high (4.5–6.5 DMFT), very high (> 6.6 DMFT). WHO and the World Dental Association set one of the global targets to be achieved in terms of oral and dental health as DMFT below 3 for 12 years old [39]. Archana et al. (2022) found the mean DMFT score of 6-year-old children to be 2.5 ± 2.75, while 12-year-old children had 1.2 ± 1.33 [40]. In our study, the DMFT and dft mean scores were 1.4 ± 2.23 and 5.6 ± 3.32 for the 6–9 age group, respectively; 1.9 ± 1.98 and 1.5 ± 2.14 for the 10–12 age group, respectively. According to this, it is seen that the target of WHO and the World Dental Association for permanent and deciduous teeth has been achieved in this research sample.

Education level is an important socioeconomic indicator that affects knowledge and healthy decision-making ability [41]. Highly educated families have a more positive attitude and show a stronger tendency to control their children’s sugar consumption than low-educated families [42]. It is known that the low education level of the family is a risk factor in terms of dental caries in children [43]. In the study conducted by Koçanalı et al. with 300 children between the ages of 7–13, it was found that the educational status of the mother was positively related to the frequency of tooth brushing [44]. Similar results were found in a study conducted with amateur athletes, and it was determined that the children of families with low socioeconomic status had insufficient tooth brushing habits [45]. Parallel to the studies, it was found in this study that the mean dft/DMFT indexes decreased as the mother’s education level increased. As the family’s education level increases, the awareness and consciousness level about oral and dental health increases. Since children see their parents as role models, raising awareness among parents about protecting oral health will be very important.

Caries and obesity are significant public health concerns that have an impact on numerous children and adolescents around the world. Both can have detrimental effects on health and quality of life [46]. In this study, dft and DMFT values did not differ according to BMI groups. Recent systematic reviews have also found that there is insufficient evidence to show a relationship between dental caries and obesity [27, 28]. The possible explanations for this situation may be, firstly, that the etiology of obesity and dental caries is multifactorial and that various environmental and genetic factors are effective on them. So it is impossible to explain their link with just one common risk factor. In addition, oral hygiene practices and socioeconomic status are reported to be more important risk factors for dental caries formation than obesity. Second, obesity may result from increased dietary fat consumption, which has less of an impact on dental caries development than a diet high in sugar [27].

In the study, although there were relationships between dietary calcium intake and DMFT score, dietary glucose and fructose intake, and dft score, these relationships were insignificant in the regression model. The potential cariogenicity of sucrose, glucose, and fructose is similar. Lactose is less cariogenic than other mono and disaccharides. In a study examining the relationship between the type and amount of carbohydrates taken and dental caries, it was observed that the highest quartile sucrose intake (114 g/day) increased the incidence of caries twice compared to the lowest quartile intake (69 g/day) [47]. In the same study, more than 10% contribution of sugar to energy intake and fructose consumption significantly increased the risk of caries [47]. In another study conducted in Turkish children, a positive correlation was found between glucose intake and the DMFT index [22]. Also, a positive correlation was found between dft and fructose intake. In another study, daily intake of calcium were inversely associated with primary caries index, but it was insignificant in regression analysis [48]. In this study, nutrients were not found to be risk factors for DMFT and dft scores in the regression model. This finding suggests that instead of focusing on nutrient intake alone, examining the child’s general diet quality will provide more information about the effects of nutrition on the incidence of dental caries [49].

The KIDMED questionnaire is a validated tool utilized to evaluate the level of adherence to the MD among school-aged children [35]. A study reveals a negative correlation between adherence to the MD and the prevalence, extent, and severity of caries among the pediatric population [21]. The study conducted by Inan-Eroglu et al. [7] investigated the correlation between adherence to the MD and the dental caries in a sample of 395 children. The study’s findings indicated that there was no statistically significant difference in the prevalence of decaying, filled, and missing teeth between children with low KIDMED scores and those with good or medium scores. In another study, there was no statistically significant difference in dental caries according to the KIDMED classification [22]. In this study, KIDMED score was negatively associated with DMFT score, but not dft. The primary factor contributing to different results is likely the subjective nature of the KIDMED questionnaire, which relies on the self-reporting of parents and children. Therefore, more research is needed to evaluate the relationship of the MD with dental caries. It may be effective in oral and dental health because the MD contains anticariogenic agents due to its plant-based food content. Also, MD does not include ultra-processed products, carbonated beverages, or industrial pastries with high levels of added sugars. In addition to adhering to the MD and promoting healthy eating habits, it is important to employ more strategies in order to prevent the occurrence of dental caries.

### Limitation

This study had some limitations. First, the questionnaire obtained the study data, other than food consumption records, anthropometric measurements and dental examination, which may cause subjective interpretation. Secondly, carrying out the study in a metropolitan city, the hospital where the data were collected is a private dental clinic, and therefore, children from families with higher socioeconomic levels may have been evaluated in the study. All of these may be a selection bias and limit generalisability. Finally, the food consumption record could have been taken for at least three consecutive days instead of two days. Despite some limitations, this study will contribute to the literature by examining the relationship between dental caries and nutrition in children in a multifaceted manner, including compliance with the MD, and food consumption analysis.

## Conclusion

Diet plays a major role in the prevention of dental caries, which is an important public health problem, especially in school-age children. In this study, adherence to the MD rather than nutrients was found to be important in dental caries. In addition to adherence to the MD, maternal education level was also found to be a determinant factor in dental caries in children. Further studies should be conducted to assess the impact of the MD on dental caries in children to develop dietary interventions for preventative purposes.

## Data Availability

The datasets used and/or analysed during the current study available from the corresponding author on reasonable request.

## References

[1] Kassebaum N, Bernabé E, Dahiya M, Bhandari B, Murray C, Marcenes W (2015). Global burden of untreated caries: a systematic review and metaregression. J Den Res.

[2] World Health Organization. Oral health, Key Facts. https://www.who.int/news-room/fact-sheets/detail/oral-health. Accessed 15 April 2023.

[3] Kazeminia M, Abdi A, Shohaimi S, Jalali R, Vaisi-Raygani A, Salari N, Mohammadi M (2020). Dental caries in primary and permanent teeth in children’s worldwide, 1995 to 2019: a systematic review and meta-analysis. Head Face Med.

[4] T.C. Sağlık Bakanlığı Sağlık Hizmetleri Genel Müdürlüğü. Türkiye Ağız Diş Sağlığı Profili Araştırma Raporu 2018. https://dosyamerkez.saglik.gov.tr/Eklenti/42552/0/tadsppdf.pdf. Accessed 15 April 2023.

[5] BaniHani A, Deery C, Toumba J, Munyombwe T, Duggal M (2018). The impact of dental caries and its treatment by conventional or biological approaches on the oral health-related quality of life of children and carers. Int J Paediatr Dent.

[6] Obregón-Rodríguez N, Fernández-Riveiro P, Piñeiro-Lamas M, Smyth-Chamosa E, Montes-Martínez A, Suárez-Cunqueiro MM (2019). Prevalence and caries-related risk factors in schoolchildren of 12-and 15-year-old: a cross-sectional study. BMC Oral Health.

[7] Inan E, Özsin C, Esra R, Büyüktuncer Z, Uzamis M, Güçiz B (2017). Is diet quality associated with early childhood caries in preschool children? A descriptive study. Turk J Pediatr.

[8] Morikava FS, Fraiz FC, Gil GS, de Abreu MHNG, Ferreira FM (2018). Healthy and cariogenic foods consumption and dental caries: a preschool-based cross‐sectional study. Oral Dis.

[9] Karğin D, Korkmaz BO, Mungan NC, Akyüz S (2021). Evaluation of healthy Nutrition Index-2015, Dental health and oral flora relationship in school-age children. Clin Exp Health Sci.

[10] Martínez LM (2021). Relationship between dental caries and adherence to Mediterranean diet in a population of children. Nutr Clín Diet Hosp.

[11] Mahan LK, Raymond JL (2017). Nutrition for oral and dental health. Krause’s food & nutrition therapy.

[12] World Health Organization. Guideline: sugars intake for adults and children. 2015. https://www.who.int/publications/i/item/9789241549028. Accessed 15 April 2023. 25905159

[13] Mahboobi Z, Pakdaman A, Yazdani R, Azadbakht L, Montazeri A (2021). Dietary free sugar and dental caries in children: a systematic review on longitudinal studies. Health Promot Perspect.

[14] American Academy of Pediatric Dentistry. Policy on dietary recommendations for infants, children, and adolescents. 2022. https://www.aapd.org/media/policies_guidelines/p_recdietary.pdf. Accessed 15 April 2023.

[15] Mäkinen KK (2011). Sugar alcohol sweeteners as alternatives to sugar with special consideration of xylitol. Med Princ Pract.

[16] Runnel R, Mäkinen KK, Honkala S, Olak J, Mäkinen P-L, Nõmmela R, Vahlberg T, Honkala E, Saag M (2013). Effect of three-year consumption of erythritol, xylitol and sorbitol candies on various plaque and salivary caries-related variables. J Dent.

[17] Flemming J, Meyer-Probst CT, Speer K, Koelling-Speer I, Hannig C, Hannig M (2021). Preventive applications of polyphenols in dentistry—A review. Int J Mol Sci.

[18] Ye S, Yin D, Sun X, Chen Q, Min T, Wang H, Wang L (2022). Molecular cloning, expression, and functional analysis of glycosyltransferase (TbUGGT) gene from Trapa bispinosa Roxb. Molecules.

[19] Rodríguez-Rejón AI, Castro-Quezada I, Ruano-Rodríguez C, Ruiz-López MD, Sánchez-Villegas A, Toledo E, Artacho R, Estruch R, Salas-Salvadó J, Covas MI. Effect of a Mediterranean diet intervention on dietary glycemic load and dietary glycemic index: the PREDIMED study. J Nutr Met. 2014; 2014:985373. 10.1155/2014/985373 PMC4180650 25295183

[20] Atkinson FS, Khan JH, Brand-Miller JC, Eberhard J (2021). The impact of carbohydrate quality on dental plaque pH: does the glycemic index of starchy foods matter for dental health?. Nutrients.

[21] Marqués-Martínez L, Pérez-Bermejo M, Lairón-Peris AR, Guinot-Barona C, Borrell-García C, García-Miralles E (2022). Association between the severity of dental caries and the degree of adherence to the Mediterranean diet in the pediatric population. Nutrients.

[22] İnan E, Keçeli Tİ, Özel HG, Tekçiçek M (2013). Hacettepe Üniversitesi Diş Hekimliği Fakültesi Pedodonti Kliniğine başvuran 6–12 yaşlarındaki bir grup sağlıklı çocukta beslenme durumu ve diş çürüğü ilişkisi. Bes Diy Der.

[23] Aoun E, Ballo L, Elhabony S, Arheiam A (2023). Association between dental caries and obesity among Libyan schoolchildren during the armed conflict in Benghazi. BMC Oral Health.

[24] Manohar N, Hayen A, Fahey P, Arora A (2020). Obesity and dental caries in early childhood: a systematic review and meta-analyses. Obes Rev.

[25] Hayden C, Bowler JO, Chambers S, Freeman R, Humphris G, Richards D, Cecil JE (2013). Obesity and dental caries in children: a systematic review and meta-analysis. Community Dent Oral Epidemiol.

[26] Hooley M, Skouteris H, Boganin C, Satur J, Kilpatrick N (2012). Body mass index and dental caries in children and adolescents: a systematic review of literature published 2004 to 2011. Syst Rev.

[27] Alshihri AA, Rogers HJ, Alqahtani MA, Aldossary MS. Association between dental caries and obesity in children and young people: a narrative review. Int J Dent. 2019, vol. 2019, Article ID 9105759, 10.1155/2019/9105759. 10.1155/2019/9105759 PMC6525928 31191654

[28] Paisi M, Kay E, Bennett C, Kaimi I, Witton R, Nelder R, Lapthorne D (2019). Body mass index and dental caries in young people: a systematic review. BMC Pediatr.

[29] Youssefi MA, Afroughi S. Prevalence and associated factors of dental caries in primary schoolchildren: an Iranian setting. Int J Dent. 2020;2020. 10.1155/2020/8731486. Article ID 8731486. 10.1155/2020/8731486 PMC7201520 32399035

[30] World Health Organization. Oral health surveys: basic methods. World Health Organization; 2013.

[31] Rakıcıoglu N, Acar NT, Ayaz A, Pekcan G (2009). Yemek ve Besin Fotograf Katalogu-Olçü Ve Miktarlar.

[32] Dimopoulou M, Antoniadou M, Amargianitakis M, Gortzi O, Androutsos O, Varzakas T (2023). Nutritional factors Associated with Dental Caries across the Lifespan: a review. Appl Sci.

[33] World Health Organization. (2019). Ending childhood dental caries: WHO implementation manual.

[34] BeBiS, Nutrition Data Base Software Data Base Version 8. The German Food Code and Nutrient Data Base (BLS II.3, 1999) with additions from UDSA-sr and other sources. 2004.

[35] Serra-Majem L, Ribas L, Ngo J, Ortega RM, García A, Pérez-Rodrigo C, Aranceta J (2004). Food, youth and the Mediterranean diet in Spain. Development of KIDMED, Mediterranean Diet Quality Index in children and adolescents. Public Health Nutr.

[36] Şahingöz SA, Özgen L, Yalçın E. Akdeniz Diyet Kalitesi Ölçeğinin (Mediterranean Diet Quality-KIDMED) Geçerlik ve Güvenirlik Çalışması. In: *Proceedings Book of 5th International Eurasian Congress on Natural Nutrition, Healthy Life & Sport: 2019*; 2019: 1078–1088.

[37] Lohman TG, Roche AF, Martorell R. Anthropometric standardization reference manual. Volume 177. Human kinetics books Champaign; 1988.

[38] Onis Md, Onyango AW, Borghi E, Siyam A, Nishida C, Siekmann J (2007). Development of a WHO growth reference for school-aged children and adolescents. Bull World Health Organ.

[39] Federation Dentaire Internationale/World Health Organization (1982). Global goals for oral health in the year 2000. Int Dent J.

[40] Archana SP, Gupta N, Rajmohan S (2022). Dental caries prevalence in the children of age group 6 and 12 years on the basis of various oral hygiene factors in the Himachal Population: A Cross Sectional Study. J Posit School Psychol.

[41] Laaksonen M, Rahkonen O, Karvonen S, Lahelma E (2005). Socioeconomic status and smoking: analysing inequalities with multiple indicators. Eur J Public Health.

[42] ÅstrØm A, Kiwanuka S (2006). Examining intention to control preschool children’s sugar snacking: a study of carers in Uganda. Int J Paediatr Dent.

[43] Wigen TI, Skaret E, Wang NJ (2009). Dental avoidance behaviour in parent and child as risk indicators for caries in 5-year‐old children. Int J Paediatr Dent.

[44] Koçanalı B, Ak AT, Çoğulu D (2014). Çocuklarda diş çürüğüne neden olan faktörlerin incelenmesi. Pediatr Res.

[45] Dinç BM, Tosun G (2022). Çocuk sporcularin oral hijyen alişkanliklarinin, beslenme şekillerinin, sporla ilişkili yaşam kalitelerinin ve sosyoekonomik durumlarinin değerlendirilmesi. Selcuk Dent J.

[46] Ravaghi V, Rezaee A, Pallan M, Morris AJ (2020). Childhood obesity and dental caries: an ecological investigation of the shape and moderators of the association. BMC Oral h-Health.

[47] Palacios C, Rivas-Tumanyan S, Morou-Bermúdez E, Colon AM, Torres RY, Elías-Boneta AR (2016). Association between type, amount, and pattern of carbohydrate consumption with dental caries in 12-year-olds in Puerto Rico. Caries Res.

[48] Lin H-S, Lin J-R, Hu S-W, Kuo H-C, Yang Y-H (2014). Association of dietary calcium, phosphorus, and magnesium intake with caries status among schoolchildren. Kaohsiung J Med Sci.

[49] Carvalho Silva C, Gavinha S, Vilela S, Rodrigues R, Manso MC, Severo M, Lopes C, Melo P. Dietary patterns and oral health behaviours associated with caries development from 4 to 7 years of age. Life. 2021, 11(7):609. 10.3390/life11070609 PMC8305377 34202656

